# Delta Opioid Receptor and Its Peptide: A Receptor-Ligand Neuroprotection

**DOI:** 10.3390/ijms140917410

**Published:** 2013-08-23

**Authors:** Meaghan Staples, Sandra Acosta, Naoki Tajiri, Mibel Pabon, Yuji Kaneko, Cesar V. Borlongan

**Affiliations:** Department of Neurosurgery and Brain Repair, University of South Florida College of Medicine, Tampa, FL 33612, USA; E-Mails: meaghans@mail.usf.edu (M.S.); sacosta@health.usf.edu (S.A.); ntajiri@health.usf.edu (N.T.); mpabon@health.usf.edu (M.P.); ykaneko@health.usf.edu (Y.K.)

**Keywords:** stroke, delta opioid receptors, neuroprotection, DADLE, neuronal death

## Abstract

In pursuit of neurological therapies, the opioid system, specifically delta opioid receptors and delta opioid peptides, demonstrates promising therapeutic potential for stroke, Parkinson’s disease, and other degenerative neurological conditions. Recent studies offer strong evidence in support of the therapeutic use of delta opioid receptors, and provide insights into the underlying mechanisms of action. Delta opioid receptors have been shown to confer protective effects by mediating ionic homeostasis and activating endogenous neuroprotective pathways. Additionally, delta opioid agonists such as (D-Ala 2, D-Leu 5) enkephalin (DADLE) have been shown to decrease apoptosis and promote neuronal survival. In its entirety, the delta opioid system represents a promising target for neural therapies.

## 1. Introduction

The opioid system is composed of various opioid peptides and their corresponding receptors. Opioids are a group of inhibitory neurotransmitters that are involved in a variety of functions including pain regulation and respiratory rate control. The pharmacologic effects exhibited by various opioid peptides are mediated by opioid receptors, with each receptor recognizing a unique group of opioid and non-opioid ligands. The family of classical Gi-protein coupled receptors which inhibit adenylyl cyclase, is divided into three primary subgroups: μ- (MOR), κ- (KOR), and δ-opioid receptors (DOR). Endogenous opioid peptides including the endorphins, dynorphins, and enkephalins, associate with the MOR, KOR, or DOR respectively. Opioid receptors elicit diverse pharmacologic effects depending on their opioid classification. These receptors are present throughout the central and peripheral nervous systems, as well as various peripheral organs including the heart, lungs, liver, and gastrointestinal tract [1–6]. Accumulating evidence suggests that the opioid system may confer protection against degenerative neurological diseases characterized by oxygen-, blood-, and energy depleting states [7,8]. A study conducted by Mayfield and colleagues demonstrated extended survival during hypoxia when animals were pretreated with an opioid receptor agonist [9]. Additionally, it was shown that opioid-induced protection could be inhibited by DOR antagonists, but not MOR and KOR antagonists [10]. These data suggest that the opioid system is involved in neuroprotection against hypoxic and ischemic events, and is likely mediated primarily by DOR and delta opioid peptides.

## 2. DOR: The Receptor and Neuroprotection

Accumulating evidence has demonstrated that DOR activation in response to hypoxic/ischemic stress, may afford neuroprotective effects. In the late 1980s, Xia and colleagues observed that the turtle brain has a higher density of DOR than the rat brain [11]. Furthermore, the turtle brain demonstrates a higher tolerance to hypoxic/ischemic conditions than the rat brain [12,13]. That increased DOR density in the brain lends resistance against hypoxic/ischemic injury implicates the close participation of this opioid receptor in neuroprotection. Indeed, the potential relationship between these two phenomena becomes of interest in our quest to understand the role of DOR in neuroprotection. To investigate this proposed connection between DOR activation and neuroprotection, researchers devised an experimental paradigm of neuroexcitotoxicity by adding glutamate to cultured cortical neurons [14]. Neurons which had been exposed to 100 μmol/L glutamate for 4 h daily over a period of 8–10 days showed significant neuronal injury [14]. However, activation of the DOR, via administration of (D-Ala 2, D-Leu 5) enkephalin (DADLE), decreased glutamate-induced injury by almost half [14]. Additionally, activation of MOR and KOR did not elicit any significant protective effects [14,15], suggesting that the DORs, not MORs or KORs, are responsible for the observed neuroprotective effects.

**Figure 1:**
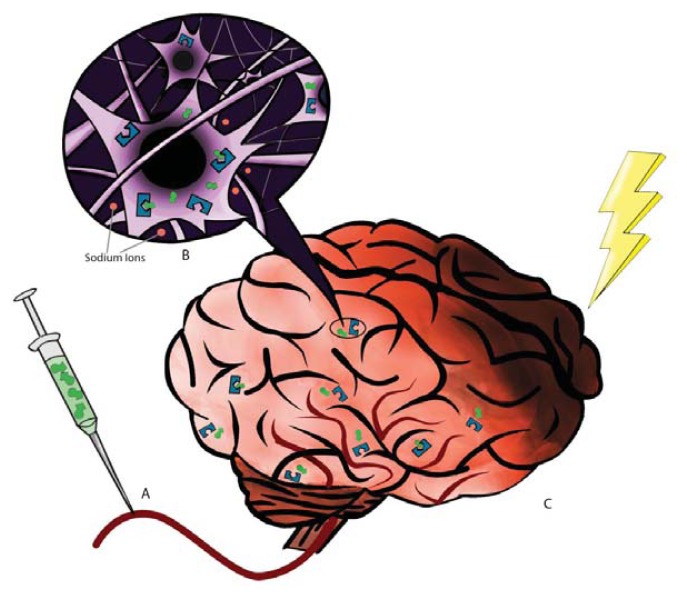
DOR-Induced Neuroprotection. (**A**) A DOR agonist such as DADLE is administered via the middle cerebral artery; (**B**) The DOR agonist binds to and activates DOR in the brain, inhibiting the influx of Na^+^ and activating the PKC/ERK pathway; (**C**) Activation of DOR results in decreased neuronal injury and death following an ischemic event.

Recent studies have also implicated DORs in ischemia [16–18]. Following middle cerebral artery occlusion (MCAO) in mice, delta binding sites decreased prior to reductions in κ- or μ-binding sites, concomitant with infarct core extension [16,19]. These *in vitro* and *in vivo* studies provide evidence that the increased DOR activation is key to suppressing glutamate-induced neurotoxicity and hypoxic/ischemic injury. These observed DOR expression patterns in the brain are likely coupled with DOR signaling pathways, altogether affording neuroprotection against injurious brain insults (Figure 1). Although the underlying signaling mechanism of DORs in neuroprotection is not fully known, recent scientific advances have been made in our understanding of these downstream pathways accompanying DOR expression patterns. Here, we discussed two major DOR-mediated mechanisms, namely Na^+^ channel and protein kinase signaling pathways. Acute hypoxic/ischemic stress causes an immediate loss of ionic homeostasis characterized by an efflux of K^+^, and an influx of Cl^−^, Na^+^, and Ca^+2^ [20,21]. This increased efflux of K^+^ is typical of hypoxia/ischemia [20,22] and can cause neuronal injury and death [23–30]. Numerous studies have demonstrated that activation of the DOR reduces K^+^ leakage following ischemia [31–35] thereby decreasing consequent neuronal death [26,27]. Furthermore, increased expression of DOR has been shown to inhibit the function of voltage-gated Na^+^ channels [21] and thus directly decreasing the influx Na^+^ and indirectly suppressing the efflux of K^+^ [21,24,34,35]. This inhibition of Na^+^ influx and the resulting neuroprotective effects of DOR were blocked following exposure to low Na^+^ perfusion, Na^+^ channel blocker TTX, and NMDA receptor channel blocker MK 801 [34,35]. Together these data suggest that inhibition of Na^+^ influx is a pivotal underlying mechanism for DOR neuroprotection against the disruption of ionic homeostasis associated with hypoxic/ischemic insult.

DOR may also elicit neuroprotective effects by mediating endogenous protein kinase signaling pathways. Activation of DORs has been shown to prevent cell death by blocking the phosphorylation of p38 via stimulation of protein kinase C (PKC) and mitogen-activated protein kinase (MAPK)-ERK1/2 [36–40]. Indeed, treatment with a PKC inhibitor has been shown to diminish DOR mediated neuroprotection against ischemia/hypoxia [36]. Using a hypoxic preconditioning (HPC) approach, DOR-mediated neuroprotection in HPC neurons were found to solicit PKC activities, in that DOR-transduced signals enhance pERK-Bcl 2 activity, but suppress those of pp38-cytochrome *c*, displaying a “yin-yang” pattern [36]. These results highlight DOR as an oxygen-sensitive protein, which enhances the intracellular activity of the G protein-PKC-pERK-Bcl 2 pathway and suppresses the pp38 and cytochrome *c* death signals [36]. This PKC-dependent pathway has also been implicated in DOR attenuation of K^+^ efflux and maintenance of ionic homeostasis [33]. Similarly, DOR as an oxygen-sensitive protein has been extended in HPC-mediated retinoprotection following intraocular pressure (IOP) elevation [38]. HPC attenuated neuronal loss and apoptosis in the IOP retina via upregulation of hypoxia-inducible factor-1alpha (HIF-1alpha) that induces the expression of DOR, and subsequently activates extracellular signal-regulated kinase resulting in anti-oxidative protection of the IOP retina [38]. In essence, Peng et al. demonstrated that an up-regulation of HIF-1alpha occurs following hypoxic preconditioning [38]. This upregulation of HIF-1alpha increases the expression of DOR which induced neuroprotection via the ERK signaling pathway [38]. Additional support of an ERK-mediated neuroprotection by DOR reveals that these DOR-induced neuroprotective effects are inhibited by treatment with an ERK inhibitor [36]. In addition to neuroprotection, DOR via the PKC signaling pathway, has also been implicated in neurogenesis [39] in that the stimulation of DOR by a selective DOR agonist promoted neural differentiation of multipotent neural stem cells, which was inhibited by treatment with a PKC inhibitor [39]. In parallel, this PKC-mediated neuroprotection via DOR activation has been seen with DADLE treatment in the severe hypoxic state of oxygen-glucose deprivation (OGD) model [40]. DADLE increased phosphorylation of ERK and prevented OGD-induced p38 phosphorylation, but no appreciable effect on phosphorylation of JNK, further highlighting that DOR-DADLE neuroprotection may be due to the dynamic balance between the activation of ERK and the p38 [40]. Altogether these data demonstrate the pivotal role of DOR mediation of PKC and ERK signaling pathways for neuroprotection. Nonetheless, further studies are warranted to elucidate DOR-mediated neuroprotection via MAPK (p38 or ERK) [36,40] and PKC signaling [38,39], especially on the involvement of genes that are either upregulated or downregulated, which should guide treatment strategies designed to highly regulate DOR expression patterns. Furthermore, additional studies are also needed to better understand why and how neuroprotective signals through DOR differ from MOR and KOR, given the fact that PKC pathways are also activated by MOR and KOR ligands [14,15].

## 3. DADLE: The Ligand and Neuroprotection

Hibernation is a unique natural model that allows animals to survive typically detrimental oxygen-, blood-, and energy-deprived conditions. For this reason, hibernation has been a particular interest for many researchers in search of potential neural therapies for disease states with similar conditions. A search for further understanding of the molecular components involved in hibernation led to the discovery that plasma from thirteen-lined ground squirrels could induce hibernation when injected into summer active ground squirrels [41]. The hibernation inducing trigger (HIT) was identified as a protein factor that co-migrates with albumin [42,43]. Due to the ability of opioids to elicit physiological conditions similar to hibernation, it has been speculated that HIT may function as an opioid; however recent evidence suggests that HIT releases endogenous opioids rather than function as one itself [42]. Alternatively, this suggests that the opioid system in general participates in achieving hibernation. Investigation into the hibernation-inducing ability of opioids and opioid receptors demonstrated that each class of opioid receptors, μ, κ, and δ, has varying potency for inducing hibernation. MOR and KOR selective antagonists, such as morphine and dynorphin, were ineffective in inducing hibernation in summer active ground squirrels [43,44]. However, the DOR agonist DADLE was highly effective in inducing hibernation [43]. As a result, several studies investigating the neuroprotective potential of the opioid system have focused on DORs and DOR selective ligands such as DADLE.

DADLE is an opioid peptide that binds primarily to DORs and it is therefore of interest when pursuing potential neuroprotective therapies. A study conducted by Tsao and colleagues investigated the effects of DADLE against the dopamine neurotoxicity of methamphetamine (METH). A high single dose of METH, or prolonged use at a medium dosage, generated long-term loss of striatal dopaminergic terminals [45]. When DADLE was administered 2 weeks after the delivery of METH, dopamine transporter (DAT) levels were restored from a loss of 30%, to normal levels [46]. Furthermore, administration of DALDE prior to exposure to METH completely inhibited, and even reversed METH-induced DAT loss [46,47]. Successive studies have demonstrated that the free radical scavenging nature of DADLE, and mediation by the DOR, are responsible for the protective effects of DADLE against METH-induced DAT loss [45].

The capacity of DADLE to protect the brain against METH-induced DAT loss has sparked investigation into DADLE’s potential for neuroprotective therapy for other neurological diseases. Specifically, DADLE has been suggested as a potential therapy for Parkinson’s disease, a neurological disease characterized by dopamine depletion. When pretreated with DADLE, adult male rats treated with 6-hydroxydopamine lesion, a dopamine depleting neurotoxin, exhibit increased survival of tyrosine hydroxylase immunoreactive cells [48,49]. Similarly, pretreatment with DADLE has been shown to increase cell viability of cultured primary rat fetal mesencephalic cells in a dose dependent manner [48,49]. In another *in vivo* study, DADLE was shown to enhance the survival of serum deprived PC12 cells [50]. While this suggests that DADLE may also involve a trophic factor mechanism, the primary pathway for DADLE’s neuroprotective action remains the participation of opioid receptors.

The neuroprotective effects of DADLE have also been extended to stroke. Studies have demonstrated that DADLE exhibits neuroprotection against ischemia reperfusion-induced brain damage following transient MCAO [51].Rats subjected to a 60 min unilateral MCAO, followed by either a 24 or 72 h reperfusion, exhibited extensive striatum infarction which was completely inhibited when DADLE was administered prior to the MCAO [51]. Furthermore, treatment with the universal opioid receptor antagonist naltrexone transiently blocked the early phase of DADLE-induced protection but was ineffective in blocking the prolonged effects [51] suggesting that opioid receptors are highly involved in the initial protective phase of DADLE, and the latter phase on an alternative mechanism of action. A recent study by Borlongan and colleagues further demonstrates the effectiveness of DADLE in protection against stroke [52]. Animals that were pretreated with DADLE prior to exposure to MCAO surgery demonstrated decreased behavioral deficits when compared to animals that received saline exclusively [52]. Furthermore, treatment with DADLE, or DALDE and an opioid blocker, had almost no detectable dehydrogenase deficient tissue (necrotic infarction) in the ischemic core [52]. In addition, DADLE has been shown to suppress p-53 mRNA expression, a marker for apoptosis characteristically associated with MCAO and stroke models [52–54]. Together, these data suggest that DADLE’s protective effects are promoted centrally. It has been suggested that DADLE may exert its protective effects by increasing the expression of GDNF, a highly selective dopamine neuron survival agent [55,56] that has been shown to protect against cerebral ischemia [57–59]. Accordingly, the increased levels of striatal GDNF following treatment with DADLE suggest that the striatal dopaminergic system may be a suitable target for DADLE in the treatment of ischemia [52].

The studies described above primarily capture several scenarios where neuroprotection is observed by DADLE treatment, which as noted in the preceding section has been implicated as a robust activator of DOR expression for neuroprotection. Accordingly, a major mechanism of action underlying DADLE neuroprotection can be ascribed to the peptide’s direct activation of DOR expression in the brain [40]. Thus, DOR-mediated downstream mechanisms involving Na^+^ channel regulation and PKC signaling pathway modulation, as discussed above, are likely to be similarly involved in the observed DADLE neuroprotection against ischemic injury [23–30,36–40]. However, we also acknowledge non-DOR mechanisms shown to be equally important neuroprotective pathways associated with DADLE treatment. In particular, we demonstrated that DADLE afford free-radical scavenging properties [45] and direct anti-necrotic and anti-apoptotic effects via neurotrophic factor (*i.e.*, GDNF) upregulation effects [52]. The direct rescue of injured cells, without the participation of DOR or its kinases, allows for a rapid neuroprotective effect, which would benefit well acute injuries such as stroke. However, with stroke now recognized as closely associated with massive secondary cell death, the need for the DOR-mediated neuroprotective mechanism would be equally therapeutic for stroke and other traditionally considered acute brain injuries with an evolving delayed neurodegeneration. To this end, DADLE has been shown to facilitate recruitment of endogenous stem cells [60]. Of note, cell based-therapy for stroke has been regarded as targeting the neurorestorative phase of the disease stage thereby allowing a wider therapeutic window for abrogating secondary cell death. The combined utilization of these neuroprotective and neurorestorative pathways will provide an optimal therapeutic outcome for DADLE-DOR-based treatments for stroke and related disorders. Studies directed at revealing the exact mechanisms of action of DADLE and DOR activation, as they relate to stroke cell death cascades, will further advance the clinical applications of this receptor-ligand neuroprotection.

## 4. Conclusions and Future Directions

Recent investigations into potential therapeutic benefits of the opioid system have led to the discovery of DOR-induced neuroprotection. Substantial accumulating evidence supports the clinical potential of DOR in treating cytotoxic, hypoxic, and ischemic neurological stress. Parallel studies on mechanisms underlying DOR neuroprotection have revealed different modes of action. Activation of DORs has been shown to stabilize ionic homeostasis and prevent ischemia-induced neuronal damage. Additionally, DORs have been shown to exert neuroprotection by inducing endogenous repair pathways. Furthermore, DADLE, a DOR ligand, has been shown to be effective in preventing neuronal injury and death in ischemia, Parkinson’s disease, and drug-induced stress. The opioid system, specifically DOR and DOR ligands, represents a new venue for developing neuroprotective therapies, warranting translational research in order to recognize their potential clinical applications. Additionally, because of the therapeutic benefits obtained with upregulation of DOR expression, more in-depth investigations into the DADLE-DOR mechanisms of action would facilitate optimization of opioid-induced neuroprotection.
